# Supplementary material for: TomoSwin3D: a Swin3D Transformer for the Identification and Classification of Macromolecules in 3D Cryo-ET Tomograms
Source: bioRxiv. 2026 Apr 21:2026.04.17.719219. Preprint. [Version 1] doi: 10.64898/2026.04.17.719219 (PMC13131780; doi:10.64898/2026.04.17.719219)
Supplement: Supplement 1 [file media-1.pdf]

TomoSwin3D: a Swin3D Transformer for the Identification and Classification of Macromolecules in 3D Cryo-ET Tomograms

Ashwin Dhakal[1,2], Rajan Gyawali[1,2], Jianlin Cheng[1,2,*]

[1] Department of Electrical Engineering and Computer Science, University of Missouri, Columbia, MO 65211, USA
[2] NextGen Precision Health, University of Missouri, Columbia, MO 65211, USA
*Corresponding author: Jianlin Cheng (chengji@missouri.edu)

Supplementary information

Supplementary Table S1: *Multiclass particle detection performance metrics of TomoSwin3D on the SHREC 2021 (synthetic) test data*

Class	GT	TP	FP	FN	Precision	Recall	F1
1s3x	122	85	104	37	0.45	0.697	0.547
3qm1	120	90	110	30	0.45	0.75	0.563
3gl1	123	92	57	31	0.617	0.748	0.676
3h84	144	132	22	12	0.857	0.917	0.886
2cg9	125	113	39	12	0.743	0.904	0.816
3d2f	140	129	11	11	0.921	0.921	0.921
1u6g	143	129	44	14	0.746	0.902	0.816
3cf3	139	134	2	5	0.985	0.964	0.975
1bxn	135	134	3	1	0.978	0.993	0.985
1qvr	127	124	2	3	0.984	0.976	0.98
4cr2	115	112	4	3	0.966	0.974	0.97
5mrc	121	120	2	1	0.984	0.992	0.988
Fiducial	11	11	0	0	1	1	1
**Macro mean**		1405	400	160	0.808	0.887	0.861

Supplementary Table S2: *Details of macromolecular complexes present in the SHREC 2021 dataset, sourced from SHREC 2021 competition.*

PDB	Name	Mol. weight (kDa)	Volume (nm³)	Area (nm²)	Sphericity	Eff. radius (nm)
1s3x	Hsp70 ATPase	42.75	90.82	109.8	0.89	2.481
3qm1	LJ0536 S106A	62.62	127.9	137.6	0.892	2.789
3gl1	Ssb1, Hsp70	84.61	196.5	191.2	0.855	3.083
3h84	GET3	158.08	347	370.9	0.644	2.807
2cg9	Hsp90-Sba1	188.73	401.2	358.4	0.734	3.358
3d2f	Sse1p, Hsp70	236.11	516	459.6	0.677	3.368
1u6g	Cand1-Cull1-Roc1	238.82	499.3	450.2	0.676	3.327
3cf3	P97/vcp	541.74	1136	745.2	0.707	4.573
1bxn	Rubisco	559.96	1021	583.4	0.84	5.25
1qvr	ClpB	593.36	1354	1063	0.557	3.821
4cr2	26S proteasome	1309.28	2675	1846	0.505	4.347
5mrc	Yeast mito ribosome	3325.59	6372	3161	0.526	6.047

Supplementary Table S3: *Protein IDs and their numbers present in the SHREC 2021 dataset, sourced from SHREC 2021 competition*

Protein	Quantity
1s3x	122
3qm1	120
3gl1	123
3h84	144
2cg9	125
3d2f	140
1u6g	143
3cf3	139
1bxn	135
1qvr	127
4cr2	115
5mrc	121
Fiducial	11

Supplementary Table S4: *Macromolecular complexes by their molecular weight in kDa, sourced from SHREC 2021 competition.*

Group	Weight	Proteins
Small	<200	1s3x, 3qm1, 3gl1, 3h84, 2cg9
Medium	200-600	3d2f, 1u6g, 3cf3, 1bxn, 1qvr
Large	600+	4cr2, 5mrc

Supplementary Table S5: *Multiclass particle detection performance metrics of TomoSwin3D on the SHREC 2020 (synthetic) test data*

Protein	GT	TP	FP	FN	Precision	Recall	Miss Rate	F1
1s3x	233	205	28	28	0.88	0.88	0.12	0.88
3qm1	241	216	23	25	0.904	0.896	0.104	0.9
3gl1	229	203	24	26	0.894	0.886	0.114	0.89
3h84	240	213	20	27	0.914	0.887	0.113	0.901
2cg9	228	220	18	8	0.924	0.965	0.035	0.944
3d2f	214	205	17	9	0.923	0.958	0.042	0.94
1u6g	217	208	13	9	0.941	0.959	0.041	0.95
3cf3	238	231	15	7	0.939	0.971	0.029	0.955
1bxn	245	238	13	7	0.948	0.971	0.029	0.96
1qvr	226	218	14	8	0.94	0.965	0.035	0.952
4cr2	231	225	10	6	0.957	0.974	0.026	0.966
4d8q	240	232	12	8	0.951	0.967	0.033	0.959
	**2782**	**2614**	**207**	**168**	**0.926**	**0.940**	**0.060**	**0.933**

Supplementary Table S6: *Details of macromolecular complexes present in the SHREC 2020 dataset, sourced from SHREC 2020 competition.*

PDB	Name	Mol. weight (kDa)	Volume (nm^3)	Area (nm^2)	Sphericity	Eff. radius (nmx)
1s3x	Hsp70 ATPase	42.75	104.1	122	0.877	2.56
3qm1	LJ0536 S106A	62.62	139.1	144.9	0.896	2.88
3gl1	Ssb1, Hsp70	84.61	207	202.6	0.835	3.065
3h84	GET3	158.08	375.3	399	0.631	2.822
2cg9	Hsp90-Sba1	188.73	394.2	380.5	0.683	3.108

3d2f	Sse1p, Hsp70	236.11	521.9	497.9	0.63	3.145
1u6g	Cand1-Cul1-Roc1	238.82	498.5	488	0.623	3.065
3cf3	P97/vcp	541.74	1123	805.7	0.648	4.181
1bxn	Rubisco	559.96	978.9	614.4	0.776	4.78
1qvr	ClpB	593.36	1255	1159	0.485	3.248
4cr2	26S proteasome	1309.28	3085	1971	0.52	4.696
4d8q	TRiC/CCT	1952.74	2152	1331	0.606	4.85

Supplementary Table S7: *Protein IDs and their numbers present in the SHREC 2020 dataset, sourced from SHREC 2020 competition*

**protein**	**Quantity**
1s3x	233
3qm1	241
3gl1	229
3h84	240
2cg9	228
3d2f	214
1u6g	217
3cf3	238
1bxn	245
1qvr	226
4cr2	231
4d8q	240
**Total**	**2782**

Supplementary Table S8: *Macromolecular complexes by their molecular weight in kDa, sourced from SHREC 2020 competition.*

Group	Weight	Proteins
Small	< 200	1s3x, 3qm1, 3gl1, 3h84, 2cg9
Medium	200 - 600	3d2f, 1u6g, 3cf3, 1bxn, 1qvr
Large	600+	4cr2, 4d8q

Supplementary Table S9: *Binary particle detection performance metrics of TomoSwin3D on the EMPIAR-10731 (experimental) test data*

**SN**	**Tomogram_Name**	**True_Count**	**TP**	**FP**	**FN**	**Precision**	**Recall**	**F1**
1	TS_01	353	311	119	42	0.723	0.881	0.794
2	TS_02	368	331	112	37	0.747	0.899	0.816
3	TS_03	320	243	111	77	0.686	0.759	0.721
4	TS_04	331	285	115	46	0.712	0.861	0.78
5	TS_05	212	195	70	17	0.736	0.92	0.818
6	TS_06	312	262	105	50	0.714	0.84	0.772
7	TS_07	305	238	91	67	0.723	0.78	0.751
8	TS_08	378	336	113	42	0.748	0.889	0.813
9	TS_09	247	204	144	43	0.586	0.826	0.686
10	TS_10	308	265	107	43	0.712	0.86	0.779

11	TS_11	221	201	77	20	0.723	0.91	0.806
12	TS_12	344	268	157	76	0.631	0.779	0.697
	Total/Mean					0.703	0.850	0.769

Supplementary Table S10: *Binary particle detection performance metrics of CFNPicker on the EMPIAR-10731 (experimental) test data*

Tomogram	True_Count	TP	FP	FN	Precision	Recall	F1
TS_01	353	282	222	71	0.56	0.799	0.658
TS_02	368	287	300	81	0.489	0.78	0.601
TS_03	320	237	172	83	0.579	0.741	0.65
TS_04	331	255	300	76	0.459	0.77	0.576
TS_05	212	153	102	59	0.6	0.722	0.655
TS_06	312	256	193	56	0.57	0.821	0.673
TS_07	305	229	270	76	0.459	0.751	0.57
TS_08	378	299	245	79	0.55	0.791	0.649
TS_09	247	180	136	67	0.57	0.729	0.639
TS_10	308	234	310	74	0.43	0.76	0.549
TS_11	221	179	146	42	0.551	0.81	0.656
TS_12	344	268	211	76	0.559	0.779	0.651
Total/Mean					**0.531**	**0.771**	**0.627**

Supplementary Table S11: *Binary particle detection performance metrics of DeepFinder on the EMPIAR-10731 (experimental) test data*

SN	Tomogram	True_Count	TP	FP	FN	Precision	Recall	F1
1	TS_01	353	268	179	85	0.6	0.759	0.67
2	TS_02	368	283	197	85	0.59	0.769	0.667
3	TS_03	320	237	158	83	0.6	0.741	0.663
4	TS_04	331	252	161	79	0.61	0.761	0.677
5	TS_05	212	153	111	59	0.58	0.722	0.643
6	TS_06	312	237	152	75	0.609	0.76	0.676
7	TS_07	305	229	159	76	0.59	0.751	0.661
8	TS_08	378	302	185	76	0.62	0.799	0.698
9	TS_09	247	180	136	67	0.57	0.729	0.639
10	TS_10	308	237	165	71	0.59	0.769	0.668
11	TS_11	221	155	127	66	0.55	0.701	0.616
12	TS_12	344	303	109	41	0.735	0.881	0.802
	Total/Mean					0.604	0.762	0.673

Supplementary Table S12: *Binary particle detection performance metrics of DeepETPicker on the EMPIAR-10731 (experimental) test data*

SN	Tomogram_Name	True_Count	TP	FP	FN	Precision	Recall	F1
1	TS_01	353	284	167	69	0.63	0.805	0.706
2	TS_02	368	286	182	82	0.611	0.777	0.684
3	TS_03	320	272	104	48	0.723	0.85	0.782

4	TS_04	331	255	123	76	0.675	0.77	0.719
5	TS_05	212	169	66	43	0.719	0.797	0.756
6	TS_06	312	239	127	73	0.653	0.766	0.705
7	TS_07	305	265	101	40	0.724	0.869	0.79
8	TS_08	378	289	122	89	0.703	0.765	0.733
9	TS_09	247	205	85	42	0.707	0.83	0.764
10	TS_10	308	238	106	70	0.692	0.773	0.73
11	TS_11	221	180	127	41	0.586	0.814	0.682
12	TS_12	344	287	160	57	0.642	0.834	0.726
	Total/Mean					0.672	0.804	0.731

Supplementary Table S13: *Protein IDs and their numbers present in the EMPIAR-10731 test dataset.*

SN	Tomogram Name	True Count of Particles
1	TS_01	353
2	TS_02	368
3	TS_03	320
4	TS_04	331
5	TS_05	212
6	TS_06	312
7	TS_07	305
8	TS_08	378
9	TS_09	247
10	TS_10	308
11	TS_11	221
12	TS_12	344

Supplementary Table S14: *Multiclass particle detection performance metrics of TomoSwin3D on the CryoETPortal test data*

**CryoETPortal_model_24**							
**Protein_Name**	**True_Count**	**TP**	**FP**	**FN**	**Precision**	**Recall**	**F1**
1dwn	26	25	4	1	0.862	0.962	0.909
1fa2	53	48	29	5	0.623	0.906	0.738
1fha	194	182	74	12	0.711	0.938	0.809
5a1a	27	26	8	1	0.765	0.963	0.852
6qzp	142	139	44	3	0.76	0.979	0.855
7n4y	40	38	12	2	0.76	0.95	0.844
**TOTAL**	**482**	**458**					**0.835**
**CryoETPortal_model_25**							
**Protein_Name**	**True_Count**	**TP**	**FP**	**FN**	**Precision**	**Recall**	**F1**
1dwn	27	25	2	2	0.926	0.926	0.926
1fa2	62	55	24	7	0.696	0.887	0.78
1fha	193	182	80	11	0.695	0.943	0.8
5a1a	34	32	4	2	0.889	0.941	0.914
6qzp	141	138	28	3	0.831	0.979	0.899
7n4y	41	40	7	1	0.851	0.976	0.909
**TOTAL/mean**	**498**	**472**					**0.871**

CryoETPortal_model_26							
**Protein_Name**	**True_Count**	**TP**	**FP**	**FN**	**Precision**	**Recall**	**F1**
1dwn	24	23	4	1	0.852	0.958	**0.902**
1fa2	68	63	26	5	0.708	0.926	**0.803**
1fha	193	187	65	6	0.742	0.969	**0.84**
5a1a	45	43	10	2	0.811	0.956	**0.878**
6qzp	140	137	31	3	0.815	0.979	**0.89**
7n4y	41	40	11	1	0.784	0.976	**0.87**
**TOTAL/mean**	**511**	**493**					**0.864**

Supplementary Table S15: *Protein name, corresponding PDB ID and their weight in CryoETPortal dataset.*

Name	PDB ID	Weight
cytosolic_ribosome	6qzp	4300
pp7_vlp	1dwn	3400
thyroglobulin	7n4y	660
beta_galactosidase	5a1a	540
ferritin_complex	1fha	450
beta_amylase	1fa2	268
